# Cost-utility analysis of treating mild stage normal tension glaucoma by surgery in China: a decision-analytic Markov model

**DOI:** 10.1186/s12962-024-00523-6

**Published:** 2024-02-12

**Authors:** Di Song, Liwen Wang

**Affiliations:** 1 The First People’s Hospital of Huzhou, The First Affiliated Hospital of Huzhou Teacher College, Huzhou, Zhejiang China; 2 Department of Ophthalmology, Huzhou Central Hospital, Affiliated Central Hospital Huzhou University, No. 1558, Sanhuan North Road, Wuxing District, Huzhou, 313000 Zhejiang China

**Keywords:** Cost-utility analysis, Normal-tension glaucoma, Trabeculectomy, Markov model

## Abstract

**Background:**

Many individuals suffer from normal tension glaucoma (NTG) in China. This study utilized Markov models to evaluate the cost-utility of applying many medications and surgery for mild-stage NTG when disease progression occurred at a mild stage.

**Methods:**

A 10-year decision-analytic Markov model was developed for the cost-utility analysis of treating mild-stage NTG with surgery and increased application of medication. We hypothesized that all 100,000 samples with a mean age of 64 were in mild stages of NTG. Transitional probabilities from the mild to moderate to severe stages and the basic parameters acquired from the CNTGS were calculated. Incremental cost-utility ratios (ICUR) were calculated for treating all patients with NTG by probabilistic sensitivity analysis (PSA) and Monte Carlo simulation. One-way sensitivity analysis were conducted by adjusting the progression rate, cost of medications or trabeculectomy, cost of follow-up, and surgical acceptance rate.

**Results:**

The ICUR of treating mild stage NTG with medication over 10 years was $12743.93 per quality-adjusted life years (QALYs). The ICUR for treating mild stage NTG patients with a 25% and 50% surgery rate with medication were $8798.93 and $4851.93 per QALYs, respectively. In this model, the cost-utility of treating NTG was sensitive to disease progression rate, surgical treatment rate, and medication costs.

**Conclusions:**

According to the results of the cost-utility analysis, it was a reasonable and advantageous strategy to administer a lot of medication and surgery for NTG in the mild stages of the disease. In the model, the greater the probability of patients undergoing surgery, the strategy becomes more valuable.

**Supplementary Information:**

The online version contains supplementary material available at 10.1186/s12962-024-00523-6.

## Background

Glaucoma is the primary cause of irreversible blindness worldwide, affecting approximately 76 million people by 2020 and over 111 million by 2040 [1]. The prevalence of glaucoma varies between 2.3 and 3.6% in the Chinese population [2]. In China, primary glaucoma is estimated to affect 9.4 million people aged 40 years and above, with 5.2 million blind in at least one eye and 1.7 million blind bilaterally [3]. Primary open-angle glaucoma (POAG) is the most common type prevalent in Africans and Caucasians [4]. However, several population-based studies demonstrated that the age-adjusted rate of Chinese POAG was similar to that of Western countries [5].

Normal tension glaucoma (NTG), which is generally defined as individuals with glaucomatous optic nerve cupping and field loss accompanied by normal intraocular pressure (IOP) [6], constitutes a significant proportion of POAG [7]. The Collaborative Normal-Tension Glaucoma Study (CNTGS), a large multicenter clinical trial, reported that a 30% IOP reduction could delay the visual field (VF) progression of NTG [8]. The Low-pressure Glaucoma Treatment Study (LoGTS) reported that VF loss was significantly less likely to occur in treated NTG [9]. Currently, IOP reduction is the predominant strategy for delaying the progression of NTG. In the United States, the total annual healthcare costs for one POAG patient range from $1570 to $2070 [10], and the direct medical costs for glaucoma exceeded $2.9 billion [11]. Glaucoma imposes a significant economic burden on families and society and reduces the patient’s quality of life [12].

According to Tang’s study [13], medications were assumed to be prescribed to patients with mild POAG in China. Patients with severe or moderate POAG were assumed to be treated by trabeculectomy. This was the current method of treatment. The average cost of initial treatment and follow-up for treatment for mild stage POAG was $256, while for moderate and severe stages, it was $345 and $230. In our study, this treatment method was regarded as the traditional method.

According to the CNTGS study, a 30% initial sustained baseline IOP reduction is a preferable treatment approach for minimizing costs and disease development, and medication and surgery are the primary methods for decreasing the IOP treatment by 30%. Patients are more willing for medication than surgery in the Chinese population. A substantial amount of medication was required to maintain a 30% reduction in baseline IOP. Nonetheless, medication adherence was insufficient to control the progression of NTG [14]. Although surgery cost much more in the first year, the therapeutic efficacy was satisfying and long-lasting [15].

In our study, the intervention consisted of administering several medications and undergoing surgery for NTG when the disease progresses to a mild stage, which was defined as a positive treatment method. To improve patient’s quality of life and balance the use of healthcare resources, the Markov model for analytical cost-utility analysis (CUA) was used to compare the positive treatment approach with the conventional treatment approach. Utility values can be used to calculate quality-adjusted life years (QALYs), which quantitatively represent the patient’s quality of life. The Markov model is a common model to perform CUA for decision-making [13].

In the Chinese population, no cost-utility analysis has demonstrated whether treating NTG by decreasing IOP by 30% from baseline was reasonable. For developing health care policy and allocating health care resources for glaucoma management, consideration of the economic burden of NTG and its impact on the quality of life and the treatment strategy for NTG was crucial. The purpose of this study was to use the Markov model to conduct a CEA of positive treatment methods, particularly trabeculectomy in China.

## Methods

**Fig. 1:**
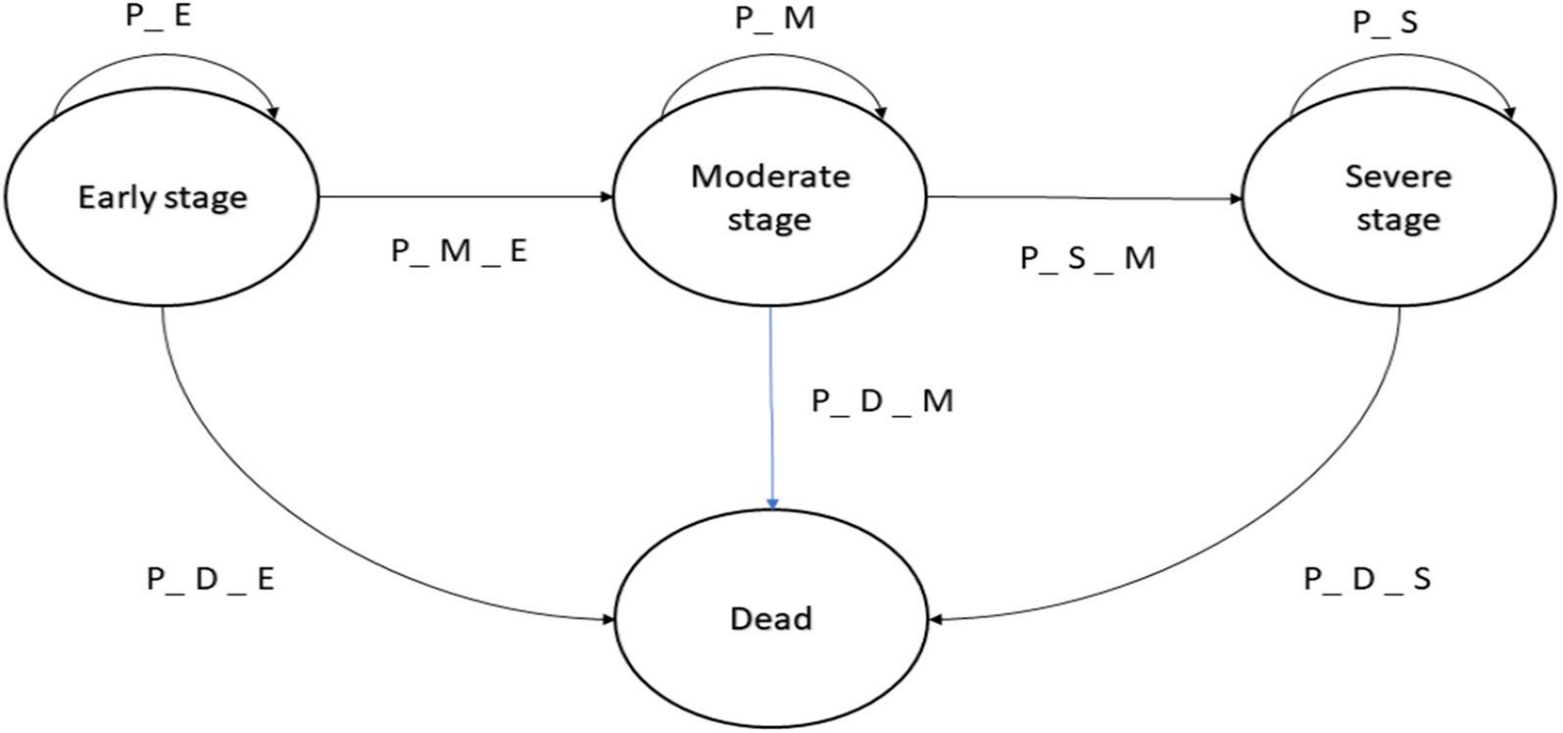
Influence diagram for glaucoma progression from mild to severe stages with death

Because the probability of progression over 10 years was projected from the CNTG study results[16], in this study, a 10-year Markov decision model was developed using Excel (Microsoft, 2019). We hypothesized that all 100,000 NTG samples aged 64 were in the mild stage of the disease. In this model, every subject in the cohort was enrolled with progression risks, medical-related costs, and the benefits associated with each treatment, and their health status changed as the disease progressed. The model included three stages of NTG, ranging from mild to moderate to severe, and each stage was associated with mortality, as shown in Fig. 1. The cycle stage of the model was set at one year, and Monte Carlo simulation, random sampling, and trials were performed. Cost and quality-adjusted life years (QALYs) were calculated for each patient as the disease progressed through this model. A half-cycle [] correction was applied to both costs and benefits [17]. In this study, 100,000 samples were run for each path in the Markov cycle tree for Probabilistic sensitivity analysis [13, 18]. According to Lee et al., the surgical rates in POAG ranged from 28.4% to 34.9% [19], the percentage of treating NTG by trabeculectomy instead of medication therapy was estimated to be between 25 and 50%. Considering the surgical failure rate and the fact that some postoperative patients require medication and maintenance, we set PSA running with five times and ten times the surgery cost improves the model’s fault tolerance.

The Incremental cost-utility radios (ICUR) calculation formula is as follows:

$$ICUR = \frac{\Delta Cost}{{\Delta Utility}} = \frac{{Cost_{Positive treatment} - Cost_{Traditional treatment} }}{{Utility_{Positive treatmen} - Utility_{Traditional treatmen} }}$$

ICUR was the primary outcome of comparing the two treatments (traditional and positive treatment methods, as indicated in the introduction) used in this study. And ICUR was later compared against the countries’ willingness to pay threshold, set at 1 GDP per capita. The per capita gross domestic product (GDP) for China was estimated to be $12692.90 in 2023, according to China’s statistical data. If ICUR was less than GDP, the favorable treatment approach was considered worthwhile. If ICUR was less than three times GDP, the positive treatment option was evaluated for clinical application [20].

### Ethical statement

This article compiled published articles for data analysis and does not include any studies involving humans/animals/plants. Therefore, informed consent was waived after institutional review board (IRB) approval. This study was a secondary analysis of data from other studies (references 20, 24, 42) that are publicly available, and it did not require ethical approval.

### Markov model: medical aspect

As POAG is a long-term, chronic disease, no study directly reported the transition probability of each stage every year. However, according to the findings of CNTGs [16], the 5-year progression rate for patients receiving mild treatment (aimed to lower IOP by 30% from initial) was 20%. At five years, the progression rate of NTG was 20% in the treated group and 60% in the observation group, similar to the 66% reported in Rei’s study [21] for Japanese patients. Therefore, we can calculate transition probability for each stage every year based on the formula rate: Multi-year probabilities to rates via the formula rate =  *− Ln (1—t-year probability) / t years*, *and then calculate the 1-year probabilities through 1-year probability* = *e(− rate ∗ 1), where t* > *1*, we determined that the 1-year progression rate was 4%. Then, we set the baseline MD score to be approximately − 5.9 dB, and the mean MD slope of progression was 0.9 dB/y [22]. After that, the transition probability matrix from mild to moderate and moderate to severe stages can be obtained over 10 years (Table 1). The final application of the formula rate of multi-year probabilities was to obtain the transition rate for different stages every year; it was 8.5%/y for mild to moderate and 3.5%/y for moderate to severe. Mild, moderate, and severe stages were defined by Humphrey mean deviation (MD) values between − 0.01 to − 6 dB, − 6.01 to − 12 dB, and − 12.01 to − 20 dB, respectively [23]. This represented the transition probability of the treatment for lowering IOP by 30% from the initial level.

**Table 1:** Estimates for utility, mortality, and other parameters

Parameters	Value	Source
Transition probabilities yearly		
Risk of progression from mild to moderate stage in observation	0.149	Tang et al. [23]
Risk of progression from moderate to severe in treatment B	0.056	Tang et al. [23]
Risk of progression in treatment A at 5 years	0.200	CNTGS [19]
Risk of progression from mild to moderate stage in treatment A	0.044	tertiary hospital
Risk of progression from moderate to severe stage in treatment A	0.018	tertiary hospital
Utility score		
Mild NTG	0.80	Tang et al. [23]
Moderate NTG	0.75	Tang et al. [23]
Severe NTG	0.71	Tang et al. [42]
Natural Mortality rates by age group, years		
65—69	0.364%	Tang et al. [23]
70—74	0.518%	Tang et al. [23]
Increased mortality risk for different groups, odds ratio		
People with mild, moderate, or severe POAG	1.8	Tang et al. [23]

The time horizon set for modeling was set at 10 years. The frequency of continuous follow-up visits was recommended by the American Academy of Ophthalmology Preferred Practice Pattern [24]. When patients were in the mild stage, both the treatment and observation groups had two follow-up visits, including a Visual field test (VF), Optical Coherence Tomography (OCT), Disc photography (DP), Slit-lamp bio-microscopy and Non-contact Tonometer, and three follow-up visits (including VF, OCT, and DP) when the disease progressed and in the remaining years.

To calculate quality-adjusted life years (QALYs), utilities for each glaucoma stage were estimated. The utility was a preference-based measure of the quality of life-related to a healthy state. Following the Hodapp Anderson-Parrish (HAP) classification criteria, stage utilities were presumed to be 0.80 for people with mild POAG, 0.75 for those with moderate POAG, and 0.71 for those with severe POAG [25].

The plan for traditional treatment method was a follow-up for mild NTG. Patients with moderate or severe NTG were presumed to be treated with trabeculectomy and postoperative medications for six weeks. Among them, 20% were assumed to fail the surgery and require long-term topical medical therapy, and the mild-to-moderate rate was 14.9%/y, and the moderate-to-severe was 5.6%/y, according to Tang’s study [13]. According to the findings of Cheng’s study, timolol and latanoprost were the two most effective IOP-lowering agents in NTG patients, assuming that patients would need dual therapy and some would require triple therapy to achieve target IOP [26, 27]. As per the Ocular Hypertension Treatment Study, the ratio of patients who were prescribed two (timolol and latanoprost) vs. three (Azopt, latanoprost, and Alphagan) medicines was 3:1 [18, 28] For the rest of the years, if the disease progresses persistently, 1.5 times of the normal dosage of medicine would be prescribed. For surgery, trabeculectomy, as the classic surgery [15] was chosen as the optimal method. Patients whose IOP were unstable after surgery would receive two (timolol and latanoprost) or three (Azopt, latanoprost, and Alphagan) medicines assumed 3:1. All costs for medication, surgery, and examination were collected in Chinese yuan but converted into US dollars using the 2021 China Statistical Yearbook exchange rate of 7.04 yuan per dollar.

### Markov model: economic aspect

This study only considered the direct costs from the payer’s perspective. The input included diagnostic tests, medication, and trabeculectomy. The costs for the medications and surgeries (Table 1) were obtained from the tertiary hospital-Huzhou Eye Hospital. These costs are regulated by the Chinese Government and vary little among institutions within the same tier of the healthcare system. Annual medication consumption was estimated based on an analysis by Rylander [29], which included the number of drops per milliliter and common dosing patterns. All costs were given in US dollars using the average 2023 exchange rate (1 US dollar = 7.04 RMB) [30]. Following the National Institute for Health and Care Excellence (NICE) recommendations [31], all costs were discounted at a rate of 3.5% per year, and utility was discounted at the same rate. Major input parameters of the current Markov Model are listed in Table 1, and all the costs of NTG patients are summarized in Table 2. Because the cost of initial and consecutive treatment was acquired from Tang’ study in 2019 [13], we recalculated the cost up to 2023 at 3.5% increase rate per year.

**Table 2:** Costs of Treatment in Clinical Management of Normal Tension Glaucoma

Cost input	US Costs in Nominal US Dollars as of 2023	Source
Treatment A		
The annual cost of dual therapy*	307.24	Tertiary hospital
The annual cost of triple therapy†	470.45	Tertiary hospital
Cost of trabeculectomy	530.97	Tertiary hospital
Cost of follow-up in no progression patients	79.55	Tertiary hospital
Cost of follow-up in progress patients	119.32	Tertiary hospital
Treatment B		
First year cost of moderate stage	381.84	Tang et al. [23]
First year cost of severe stage	381.84	Tang et al. [23]
Cost of the moderate stage in consecutive years of follow-up	254.56	Tang et al. [23]
Cost of the severe stage in consecutive years of follow-up	254.56	Tang et al. [23]

^*^ Annual cost of dual therapy was the sum of the mild costs of timolol and latanoprost

^†^ Annual cost of triple therapy was the sum of the annual cost of dual therapy plus the mild cost of a third medication, which was taken as the average mild cost of Azopt, Trusopt, and Alphagan-P

### Statistical analysis

We set the surgery rate at 50% and then performed one-way analyses for the Markov model by varying the vital input in a positive treatment method. Based on the complication or failure rate of surgery and medication, ± 20% change cost was calculated. The probability of disease state transfer in response to positive treatment was unstable, with a calculated change rate of ± 50%. [21] Probabilistic sensitivity analysis was also performed. As the standard deviation for a cost input, utility, and translation rate was missing, we assumed that 10% of the standard deviation was based on the mean value. The presumed variation range and distributions for transitional probabilities, cost, and utility are shown in Additional file 1: Appendix 1, 2 and 3.

## Results

**Fig. 2:**
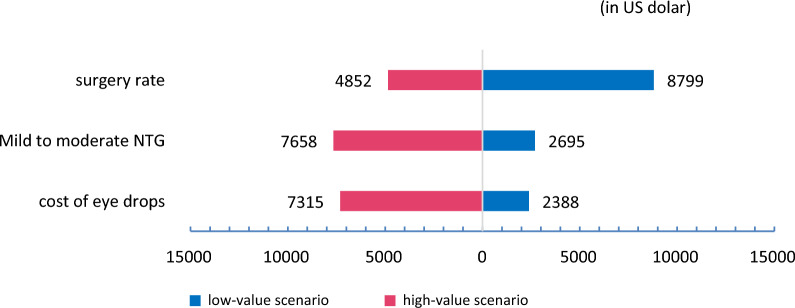
Tornado plot of 10-year accumulated incremental costs of the effectiveness of one-way sensitivity analysis. Low-value scenario: Surgery rate 25%; Transfer probability from mild to moderate NTG decrease 50%; Cost of eye drops decrease 20%. High-value scenario: Surgery rate 50%; Transfer probability from mild to moderate NTG increase 50%; Cost of eye drops increase 20%

The ICUR of treating mild stage NTG with medication over 10 years was $12743.93 per quality-adjusted life years (QALYs). The ICUR for treating mild stage NTG patients with a 25% and 50% surgery rate with medication were $8798.93 and $4851.93 per QALYs, respectively. The results of one-way sensitivity analyses at 50% surgery rate with medication strategy are shown in Table 3, and Fig. 2 displays the top three related factors for this model. Table 4 represents the PSA results of running the model with 5 and 10 times the surgery cost, the ICRU with surgery rate set at 25% were $10567.80 and $12780.13, respectively. When the surgery rate was set at 50%, the ICRU were $8391.67 and $12816.40, respectively.

**Table 3:** Basic result of ICUR and One-way sensitivity analysis for 50% surgery rate

Treatment strategy	Average costs per	Incremental cost	Average Efficacy	Incremental QALYs	ICUR, US$/QALYs
	person, $		QALYs		
Treatment all by medication					
Positive Treatment	3887.38		6.58		
Traditional Treatment	1975.79	1911.59	6.43	0.15	12,743.93
50% surgery rate					
Positive Treatment	2703.58		6.58		
Traditional Treatment	1975.79	727.79	6.43	0.15	4851.93
25% surgery rate					
Positive Treatment	3295.48		6.58		
Traditional Treatment	1975.79	1319.69	6.43	0.15	8798.93
One-way sensitivity analysis for 50% surgery rate					
Positive Treatment (transfer probability)					
Mild NTG to moderate NTG (+ 50%)	2894.83		6.55		
	1975.79	919.04	6.43	0.12	7658.67
Mild NTG to moderate NTG (-50%)	2487.89		6.62		
	1975.79	512.1	6.43	0.19	2695.26
Moderate NTG to severe NTG (+ 50%)	2703.62		6.58		
	1975.79	727.83	6.43	0.15	4852.20
Moderate NTG to severe NTG (-50%)	2703.53		6.58		
	1975.79	727.74	6.43	0.15	4851.60
Cost input for Positive Treatment					
Cost of eye drops (+ 20%)	3073.11		6.58		
	1975.79	1097.32	6.43	0.15	7315.47
Cost of eye drops (-20%)	2334.04		6.58		
	1975.79	358.25	6.43	0.15	2388.33
Cost of trabeculectomy (+ 20%)	2730.12		6.58		
	1975.79	754.33	6.43	0.15	5028.87
Cost of trabeculectomy (-20%)	2677.03		6.58		
	1975.79	701.24	6.43	0.15	4674.93
Follow-up (+ 20%)	2848.21		6.58		
	2144.06	704.15	6.43	0.15	4694.33
Follow-up (-20%)	2558.94		6.58		
	1807.53	751.41	6.43	0.15	5009.40

**Table 4:** The results of Probabilistic sensitivity analysis of different treatment strategies

Treatment strategy	Average costs	Incremental cost	Average Efficacy	Incremental QALYs	ICUR, US$/QALYs
	person, $		QALYs		
Treatment all by medication					
Positive Treatment	3973.28		6.58		
Traditional Treatment	1898.85	2074.43	6.43	0.15	13,829.53
50% surgery rate					
Positive Treatment	2765.48		6.58		
Traditional Treatment	1898.82	866.66	6.43	0.15	5777.73
25% surgery rate					
Positive Treatment	3370.94		6.58		
Traditional Treatment	1899.24	1471.70	6.43	0.15	9811.33
25% surgery rate + 5 times the cost of surgery					
Positive Treatment	3560.96		6.58		
Traditional Treatment	1975.79	1585.17	6.43	0.15	10,567.80
25% surgery rate + 10 times the cost of surgery					
Positive Treatment	3892.81		6.58		
Traditional Treatment	1975.79	1917.02	6.43	0.15	12,780.13
50% surgery rate + 5 times the cost of surgery					
Positive Treatment	3234.54		6.58		
Traditional Treatment	1975.79	1258.75	6.43	0.15	8391.67
50% surgery rate + 10 times the cost of surgery					
Positive Treatment	3898.25		6.58		
Traditional Treatment	1975.79	1922.46	6.43	0.15	12,816.40

## Discussion

In NTG, decreasing IOP was significantly more strongly associated with progressive axonal loss and retinal ganglion cell damage [18]. In a natural disease state, the progression rate and visual field loss are low; consequently, many patients do not consider this condition seriously enough to seek treatment. However, without appropriate and long-term treatment, the disease progressed more rapidly in 60% of patients compared to 20% of those who received treatment [22]. Consideration of the economic burden of NTG and its impact on quality of life was crucial for the development of health care policy and allocation of health care resources for glaucoma management. The purpose of this study was to use the Markov model to perform CUA of positive treatment methods, particularly trabeculectomy in China.

Our study showed an incremental cost-utility ratio (ICUR) of $12743.93 per QALYs when comparing the positive treatment method by medication to the conventional treatment method. The ICUR for treating NTG patients with a 25 and 50% rate for surgery with medication was $8798.93 and $4851.93 per QALYs, respectively, indicating that increasing the rate of trabeculectomy therapy would be a very worthwhile strategy, consistent with the findings of the previous study [32]. Specifically, in Li’s model [18], it was cost-effective to treat NTG patients with a mild treatment (medication or surgery). Considering the gradual progression of NTG, medication is more expensive, whereas trabeculectomy ensures greater efficacy [33] while avoiding the need for long-term treatment, thereby reducing cost. Similar to Li’s study, the cost-effectiveness decision in our models for NTG was sensitive to the progression of NTG, the costs of eyedrops, trabeculectomy, and follow-up, along with the trabeculectomy rate [18]. At the same time, we set up 5 and 10 times the cost of surgery into the model, and the results still indicated that surgery is a worthwhile choice strategy. This indicated that the positive treatment method, especially surgery in our model, has a very high fault tolerance in the event of surgical failure or subsequent treatment.

Surgery would be recommended for glaucoma patients. However, medication seems convenient for patients. According to research, the number of medications, their prolonged use, and exposure to preservatives are risk factors for the development of ocular surface disease in glaucoma patients [34]. According to Wiafe’s report [32], considering the lack of resources and competing for opportunity costs, lifelong medical therapy was impractical. Additionally, medicines were mainly found in pharmacies in large cities, so patients in rural or remote areas could not procure them easily. Medication adherence was unsatisfactory (about 20%) in a developed country [14]. It was reported that the daily cost of an eye drug such as latanoprost was US $0.87 in the developed world [35] and possibly even more in developing countries. Surgery was the cost-effective treatment for the mild stage of glaucoma patients [36]. The cost of surgery to reduce intraocular pressure has decreased over time. The cost is higher during the first three years, but when considering the number of years people live and the cost of drugs for those years, surgery is a relatively worthwhile treatment option in developing countries.

As it was a simulated study and with the analogy to many other cost-effectiveness analyses, there are several limitations to our model. Firstly, only direct costs were considered. Indirect costs such as patient’s time spent attending follow-up appointments and lost productivity due to time off were not considered. However, we can disregard this because our model set the age at 64. Additionally, patient compliance was not considered in this study, which would affect the medication cost. However, this had little effect on our model because our results indicated that surgery may be more effective.

## Conclusions

This was the first study to provide economic evidence on how to treat Chinese NTG patients cost-effectively. A better understanding of risk factors and treatment options for glaucoma patients would be helpful in improving patient’s health-related quality of life and optimizing resource allocation.

### Supplementary Information

**Additional file 1: Appendix S1.** Variation range and distributions assumed for the transitional probabilities between treatment A and treatment B. **Appendix S2.** Variation range and distributions assumed for Treatment A and B cost. **Appendix S3.** Variation range and distributions assumed for utilization. **Appendix S4.** Costs of Treatment in Clinical Management of Normal Tension Glaucoma. **Appendix S5.** Estimates for utility, mortality, and other parameters.

## Data Availability

The datasets used and/or analyzed during the current study are available from the corresponding author upon reasonable request.

## Abbreviations

NTG: Normal tension glaucoma
VFL: Visual field loss
IOP: Intraocular pressure
CNTGS: Collaborative normal-tension glaucoma study group
ICUR: Incremental cost-utility ratios
PSA: Probabilistic sensitivity analysis
QALYs: Quality-adjusted life years
POAG: Primary open-angle glaucoma
LoGTS: Low-pressure glaucoma treatment study
VF: Visual field
OCT: Optical coherence tomography
DP: Disc photography
