# Supplementary material for: Cost-utility analysis of treating mild stage normal tension glaucoma by surgery in China: a decision-analytic Markov model
Source: Cost Eff Resour Alloc. 2024 Feb 12;22:13. doi: 10.1186/s12962-024-00523-6 (PMC10863271; doi:10.1186/s12962-024-00523-6)
Supplement: Supplementary file 1 — Additional file 1: Appendix S1. Variation range and distributions assumed for the transitional probabilities between treatment A and treatment B. Appendix S2. Variation range and distributions assumed for Treatment A and B cost. Appendix S3. Variation range and distributions assumed for utilization. Appendix S4. Costs of Treatment in Clinical Management of Normal Tension Glaucoma. Appendix S5. Estimates for utility, mortality, and other parameters. [file 12962_2024_523_MOESM1_ESM.docx]

| **Transitions** | **Treatment A** | | | |
| --- | --- | --- | --- | --- |
|  | **Prob.** | **Source** | **Range for sensitivity analysis** | **Distributions used in the probability sensitivity analysis** |
| Mild NTG to moderate NTG | 4.4% | CNTGS | 4.4% ± 10% | Beta (95.56,2076.17) |
| Moderate NTG to severe NTG | 1.8% | CNTGS | 1.8% ± 10% | Beta (98.18, 5356.37) |
| **Transitions** | **Treatment B** | | | |
|  | **Prob.** | **Source** | **Range for sensitivity analysis** | **Distributions used in the probability sensitivity analysis** |
| Mild NTG to moderate NTG | 14.9% | Tang et al [] | 14.9% ± 10% | Beta (84.95, 485.19) |
| Moderate NTG to severe NTG | 5.6% | Tang et al [] | 5.6% ± 10% | Beta (94.34, 1590.37) |

**Appendix 1. Variation range and distributions assumed for the transitional probabilities between treatment A and treatment B.**

CNTGS: Group CN-TGS. Comparison of Glaucomatous Progression Between Untreated Patients With NormalTension Glaucoma and Patients With Therapeutically Reduced Intraocular Pressures. Am J Ophthalmol 1998; 126:487–497.

Tang et al : Tang J, Liang Y, O'Neill C, Kee F, Jiang J, Congdon N. Cost-effectiveness and cost-utility of population-based glaucoma screening in China: a decision-analytic Markov model. Lancet Global Health 2019; 7:968-978.

**Appendix 2. Variation range and distributions assumed for Treatment A and B cost.**

| **Items/year** | **Value Used ($)** | **Source** | **Range for sensitivity analysis($)** | **Distributions used in the probability sensitivity analysis** |
| --- | --- | --- | --- | --- |
| cost of two drops | 307.24 | tertiary hospital | 307.24 ± 10% | Gamma (100,3.14) |
| cost of three drops | 470.45 | tertiary hospital | 470.45 ± 10% | Gamma (100,4.80) |
| cost of trabeculectomy | 530.97 | tertiary hospital | 530.97 ± 10% | Gamma (100,5.42) |
| cost of follow-up for no progression | 79.55 | tertiary hospital | 79.55 ± 10% | Gamma (100,0.82) |
| cost of follow-up for progression | 119.32 | tertiary hospital | 119.32 ± 10% | Gamma (100,1.26) |
| Initial cost of treatment B for moderate | 381.84 | Tang et al | 381.84 ± 10% | Gamma (100,3.45) |
| Initial cost of treatment B for severe | 381.84 | Tang et al | 381.84 ± 10% | Gamma (100,3.45) |
| Follow cost of treatment B for moderate | 254.56 | Tang et al | 254.56 ± 10% | Gamma (100,2.30) |
| Follow cost of treatment B for severe | 254.56 | Tang et al | 254.56 ± 10% | Gamma (100,2.30) |

Tang et al: Tang J, Liang Y, O'Neill C, Kee F, Jiang J, Congdon N. Cost-effectiveness and cost-utility of population-based glaucoma screening in China: a decision-analytic Markov model. Lancet Global Health 2019; 7:968-978.

**Appendix 3. Variation range and distributions assumed for utilization.**

| **Parameters** | **Treatment A/B** | | | |
| --- | --- | --- | --- | --- |
|  | **Base-case**  **values** | **Source** | **Range for sensitivity analysis** | **Distributions used in the probability sensitivity analysis** |
| Utility for Mild NTG | 0.80 | Tang et al | 0.72 - 0.88 | Beta (19.20,4.80) |
| Utility for moderate NTG | 0.75 | Tang et al | 0.68 - 0.83 | Beta (24.25,8.08) |
| Utility for severe NTG | 0.71 | Tang et al | 0.64 - 0.78 | Beta (28.29,11.56) |

Tang et al: Tang J, Liang Y, O'Neill C, Kee F, Jiang J, Congdon N. Cost-effectiveness and cost-utility of population-based glaucoma screening in China: a decision-analytic Markov model. Lancet Global Health 2019; 7:968-978.

**Appendix 4: Costs of Treatment in Clinical Management of Normal Tension Glaucoma.**

| **Cost items** | **US Costs in Nominal US Dollars as in 2019** | **Source** |
| --- | --- | --- |
| **Treatment A** |  |  |
| Annual cost of dual therapy* | 307.24 | tertiary hospital |
| Annual cost of triple therapy† | 470.45 | tertiary hospital |
| Cost of trabeculectomy | 530.97 | tertiary hospital |
| Cost of follow-up in no progression patients | 79.55 | tertiary hospital |
| Cost of follow-up in progression patients | 119.32 | tertiary hospital |
| **Treatment B** |  |  |
| Initial cost of moderate stage | 381.84 | Tang et al |
| Initial cost of severe stage | 381.84 | Tang et al |
| Cost of moderate stage in follow-up | 254.56 | Tang et al |
| Cost of severe stage in follow-up | 254.56 | Tang et al |

* Annual cost of dual therapy was the sum of the yearly costs of timolol and latanoprost.

† Annual cost of triple therapy was the sum of annual cost of dual therapy plus the yearly cost of a third medication, which was taken as the average yearly cost of Azopt, Trusopt, and Alphagan-P.

Tang et al: Tang J, Liang Y, O'Neill C, Kee F, Jiang J, Congdon N. Cost-effectiveness and cost-utility of population-based glaucoma screening in China: a decision-analytic Markov model. Lancet Global Health 2019; 7:968-978.

**Appendix 5: Estimates for utility, mortality, and other parameters.**

| **Parameters** | **Value** | **source** |
| --- | --- | --- |
| **Transition probabilities** |  |  |
| Risk of progression from early to moderate stage in observation | 0.149 | Tang et al |
| Risk of progression from moderate to severe in treatment B | 0.056 | Tang et al |
| Risk of progression in treatment A  at 5 years | 0.200 | CNTGS |
| Risk of progression from early to moderate stage in treatment A | 0.044 | CNTGS |
| Risk of progression from moderate to severe stage in treatment A | 0.018 | CNTGS |
| **Utility score** |  |  |
| Mild NTG | 0.80 | Tang et al |
| Moderate NTG | 0.75 | Tang et al |
| Severe NTG | 0.71 | Tang et al |
| **Mortality rates by age group, years** |  |  |
| 65-69 | 0.1421% | Tang et al |
| 70-74 | 0.3149% | Tang et al |
| **Increased mortality risk for different groups, odds ratio** |  |  |
| People with mild, moderate, or severe POAG | 1.8 | Tang et al |

CNTGS: Group CN-TGS. Comparison of Glaucomatous Progression Between Untreated Patients With NormalTension Glaucoma and Patients With Therapeutically Reduced Intraocular Pressures. Am J Ophthalmol 1998; 126:487–497.

Tang et al : Tang J, Liang Y, O'Neill C, Kee F, Jiang J, Congdon N. Cost-effectiveness and cost-utility of population-based glaucoma screening in China: a decision-analytic Markov model. Lancet Global Health 2019; 7:968-978.
